# Regulation of Peroxisome Homeostasis by Post-Translational Modification in the Methylotrophic Yeast *Komagataella phaffii*

**DOI:** 10.3389/fcell.2022.887806

**Published:** 2022-04-19

**Authors:** Shin Ohsawa, Masahide Oku, Hiroya Yurimoto, Yasuyoshi Sakai

**Affiliations:** ^1^ Division of Applied Life Sciences, Graduate School of Agriculture, Kyoto University, Kyoto, Japan; ^2^ Department of Bioscience and Biotechnology, Faculty of Bioenvironmental Science, Kyoto University of Advanced Science, Kyoto, Japan

**Keywords:** methanol-induced gene expression, pexophagy, phosphorylation, WSC family proteins, MAPK cascade, ethanol repression

## Abstract

The methylotrophic yeast *Komagataella phaffii (synoym Pichia pastoris)* can grow on methanol with an associated proliferation of peroxisomes, which are subsequently degraded by pexophagy upon depletion of methanol. Two cell wall integrity and stress response component (WSC) family proteins (Wsc1 and Wsc3) sense the extracellular methanol concentration and transmit the methanol signal to Rom2. This stimulates the activation of transcription factors (Mxr1, Trm1, and Mit1 etc.), leading to the induction of methanol-metabolizing enzymes (methanol-induced gene expression) and synthesis of huge peroxisomes. Methanol-induced gene expression is repressed by the addition of ethanol (ethanol repression). This repression is not conducted directly by ethanol but rather by acetyl-CoA synthesized from ethanol by sequential reactions, including alcohol and aldehyde dehydrogenases, and acetyl-CoA synthetase. During ethanol repression, Mxr1 is inactivated by phosphorylation. Peroxisomes are degraded by pexophagy on depletion of methanol and this event is triggered by phosphorylation of Atg30 located at the peroxisome membrane. In the presence of methanol, Wsc1 and Wsc3 repress pexophagy by transmitting the methanol signal *via* the MAPK cascade to the transcription factor Rlm1, which induces phosphatases involved in dephosphorylation of Atg30. Upon methanol consumption, repression of Atg30 phosphorylation is released, resulting in initiation of pexophagy. Physiological significance of these machineries involved in peroxisome homeostasis and their post-translational modification is also discussed in association with the lifestyle of methylotrophic yeast in the phyllosphere.

## Introduction

Methylotrophic yeasts are able to grow on methanol and utilize it as their sole source of carbon and energy. This growth involves the proliferation of peroxisomes that contain the enzymes involved in methanol metabolism, such as alcohol oxidase (AOX), dihydroxyacetone synthase (DAS), as well as the cytosolic methanol-metabolizing enzymes, formaldehyde dehydrogenase (FLD), and formate dehydrogenase (FDH) (Sakai et al., 1996; Yurimoto et al., 2005). Methanol also strongly induces the transcript levels of methanol-metabolizing enzymes. These attributes make methylotrophic yeasts attractive model organisms in the study of the molecular mechanisms involved in peroxisome dynamics and as hosts for heterologous gene expression (Oku and Sakai, 2010).

Peroxisome dynamics in methylotrophic yeasts are tightly regulated by the available carbon source and this is reflected in the extent to which peroxisomal proteins are synthesized: the maximum-level of expression is achieved in the presence of methanol (methanol induction), a low-level of expression is observed in the absence of carbon source (derepression), and the synthesis is completely repressed by the presence of glucose (glucose repression) and ethanol (ethanol repression). In addition, cells regulate peroxisome biogenesis in response to changes in methanol concentration by altering the expression of methanol-induced genes (Kawaguchi et al., 2011; Ohsawa et al., 2017; Takeya et al., 2018). Methanol-induced peroxisomes are degraded by selective autophagy, i.e., pexophagy in response to methanol depletion, and strongly to the carbon source shift to glucose and ethanol (Tuttle and Dunn, 1995; van Zutphen et al., 2008; Nazarko et al., 2009; Oku and Sakai, 2016). Although several transcription factors that control methanol induction, derepression, and glucose and ethanol repression, have been identified and characterized (Leao-Helder et al., 2003; Lin-Cereghino et al., 2006; Sasano et al., 2008; Sasano et al., 2010; Sahu et al., 2014; Oda et al., 2015; Wang et al., 2016), their methanol-sensing mechanism and signaling pathways have yet to be fully elucidated.

Our recent studies on the methylotrophic yeast *Komagataella phaffii* (*synonym. Pichia pastoris*) revealed that cell-surface WSC family proteins act as methanol-sensing machineries and control the biogenesis and degradation of peroxisomes. In this review, we summarize the processes involved in the regulation of peroxisome homeostasis in the methylotrophic yeasts, including post-translational modifications such as protein phosphorylation, downstream signal cascades *via* WSC family proteins, and the molecular mechanism of ethanol repression. In addition, we discuss the physiological significance of peroxisome homeostasis for methylotrophic yeasts living in the phyllosphere environment (aerial parts of plants).

## Cell Wall Integrity and Stress Response Component Family Proteins as Methanol Sensor

Our previous studies revealed that WSC family proteins act as methanol-sensing machinery for regulating methanol-induced gene expression in *K. phaffii* (Ohsawa et al., 2017)*.* WSC family proteins were identified as “nanospring” mechanosensors of cell wall damage caused by high temperatures and hypo-osmotic stress in *Saccharomyces cerevisiae* (ScWsc1/ScSlg1) (Lodder et al., 1999; Dupres et al., 2009). In the *K. phaffii* genome*,* three WSC family protein-encoding genes have been identified (*KpWSC1, KpWSC2*, and *KpWSC3*). The significant features of Wsc family proteins, a cysteine-rich domain (CRD), a serine/threonine rich region (STR) in extracellular region, and a Rom2-interacting site at cytoplasmic tail together with a transmembrane domain (TMD) are conserved in all three KpWsc family proteins. In the *Kpwsc1∆wsc3∆* double deletion mutant, dose-dependent (0.001–1.0%) methanol-induced gene expression (*AOX, DAS, FLD, FDH*) was abolished, indicating that these WSC family proteins play a role in methanol sensing (Ohsawa et al., 2017). Characterization of KpWsc1 and KpWsc3 has revealed that KpWsc1 responds to a lower range of methanol concentration (0.01–0.05%) whilst KpWsc3 responds to a higher range (0.1–0.5%) and that KpWsc1 also acts as a sensor of cell wall damage caused by high temperature stress. These properties might enable cells to respond to a wide range of methanol concentration and to two different stimuli (methanol and high temperature stress) simultaneously. As to KpWsc2, it was not related to the regulation of methanol-inducible gene expression and high temperature stress (Ohsawa et al., 2017).

## Regulation of Peroxisome Biogenesis by Methanol

### Induction of Methanol-Induced Gene Expression by Methanol Signal from Wsc1 and Rom2 to Transcription Factors Mxr1 and Others

**FIGURE 1:**
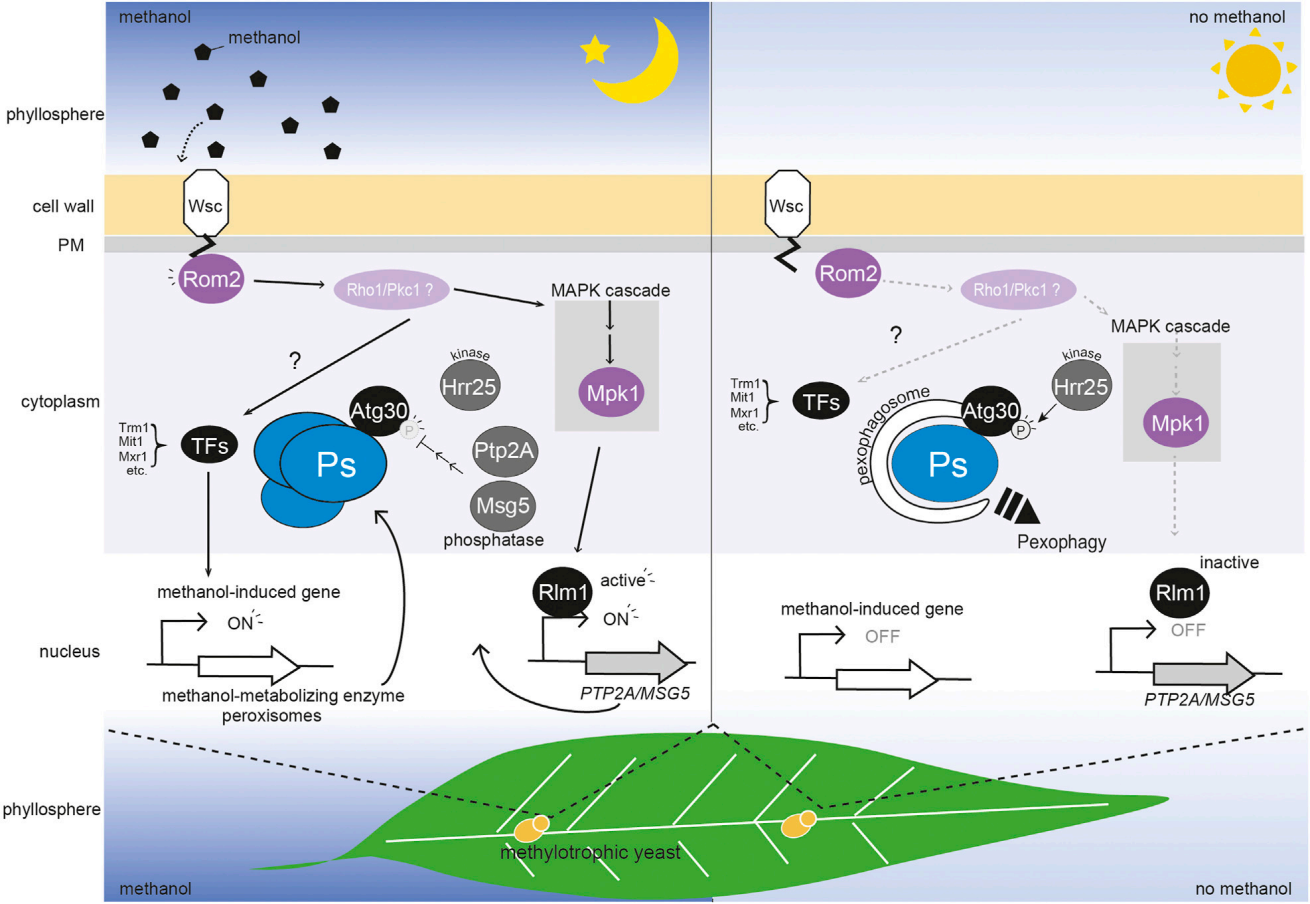
Regulation of peroxisome homeostasis by a methanol-sensing pathway mediated by WSC family proteins in the phyllosphere. WSC family proteins (Wsc1 and Wsc3) sense the change in methanol concentration, and transmit a signal to Rom2 which is then transmitted *via* two unidentified molecules (possibly Rho1 and Pkc1) to the transcription factors (TFs) Trm1, Mit1, and Mxr1. The transcription factors activate methanol-induced genes and this results in peroxisomes (Ps) biogenesis (Lin-Cereghino et al., 2006; Parua et al., 2012; Sahu et al., 2014; Wang et al., 2016; Ohsawa et al., 2018). Simultaneously, the methanol signal under Wsc1 and Rom2 is transmitted to the MAPK cascade, which includes Mpk1. The transcription factor, Rlm1 then represses pexophagy *via* expression of two phosphatases, *PTP2A* and *MSG5,* which regulate the level of phosphorylation (P) of the Atg30 pexophagy receptor. As depletion of methanol releases the biogenesis of peroxisomes and repression of pexophagy, the phosphorylation of KpAtg30 by Hrr25 is enhanced (Zientara-Rytter et al., 2018). In the phyllosphere, plant leaf methanol concentration oscillates diurnally, being higher in the dark period and lower in the light period (∼0–0.3%) (Kawaguchi et al., 2011). In the dark period **(left panel)**, methanol enhances induction of peroxisome biogenesis and represses pexophagy. In the light period **(right panel)**, methanol-induced gene expression is low, and peroxisomes are degraded *via* pexophagy.

WSC family proteins transmit signals of cell wall damage to small GTPase Rho1 by binding with the GDP/GTP exchange factor Rom2 (Philip and Levin, 2001). In *K. phaffii*, the Rom2-interacting sites of KpWsc1 and KpWsc3 are necessary for the activation of methanol-induced gene expression and for high temperature stress response. This suggests that the signal for the presence of methanol and for cell surface damage is sensed by the WSC family proteins and transmitted to KpRom2. Extensive mutagenesis analysis of KpWsc1 revealed that the KpWsc1(Y53A) mutant is deficient in methanol-induced gene expression but not in the high-temperature stress response (Ohsawa et al., 2017). Conversely, the KpWsc1(Y53F) mutant showed high sensitivity to high-temperature stress but was normal in methanol-induced gene expression. These results suggested that KpWsc1 has a distinct sensing (or signaling) mechanism for the presence of methanol and cell surface integrity. In *S. cerevisiae,* the small GTPase activates the protein kinase C (Pkc1), and then it stimulates the MAPK cascade [Bck1-Mkk1/Mkk2-Mpk1 (Slt2)]. Since KpMpk1 is unnecessary for methanol-induced gene expression (Ohsawa et al., 2021), thus the presence of methanol is unlikely to stimulate the MAPK cascade to regulate methanol-induced gene expression. We speculate that the methanol signal stimulates the MAPK-independent pathway and regulates transcription factors, such as KpMxr1, KpTrm1, and KpMit1, which are essential for methanol-induced gene expression (Lin-Cereghino et al., 2006; Parua et al., 2012; Sahu et al., 2014; Wang et al., 2016; Ohsawa et al., 2018) (Figure 1). Phosphorylation of KpMxr1 at the serine residue 215 (S215) inhibits its transcriptional activity on methanol-induced genes (Parua et al., 2012) (as described in detail below). However, it is still unknown which specific kinases phosphorylate KpMxr1. Further studies on phosphorylation of these transcription factors are needed to understand how WSC family proteins transmit the methanol signal downstream.

### Repression of Methanol-Induced Gene Expression by Glucose and Ethanol

Methanol-induced gene expression is strictly repressed by both glucose and ethanol. The transcription factor Mig1, is associated with glucose repression in methylotrophic yeasts (Stasyk et al., 2007; Zhai et al., 2012)*.* In *Ogataea polymorpha* (*synonym Hansenula polymorpha*)*,* OpMig1 is responsible for glucose repression but has a limited effect on ethanol repression (Stasyk et al., 2007) and in *Candida boidinii* and *O. methanolica* (*synonym P. pinus*) ethanol repression was observed in glucose repression-deficient mutants (Sakai et al., 1987; Sibirny et al., 1987). These findings suggest that glucose repression and ethanol repression are conducted by distinct factors. Remarkably, methanol-induced gene expression is repressed by the addition of ethanol to a methanol medium. Evidently, methylotrophic yeasts are equipped with a mechanism to discriminate between methanol and ethanol.

**FIGURE 2:**
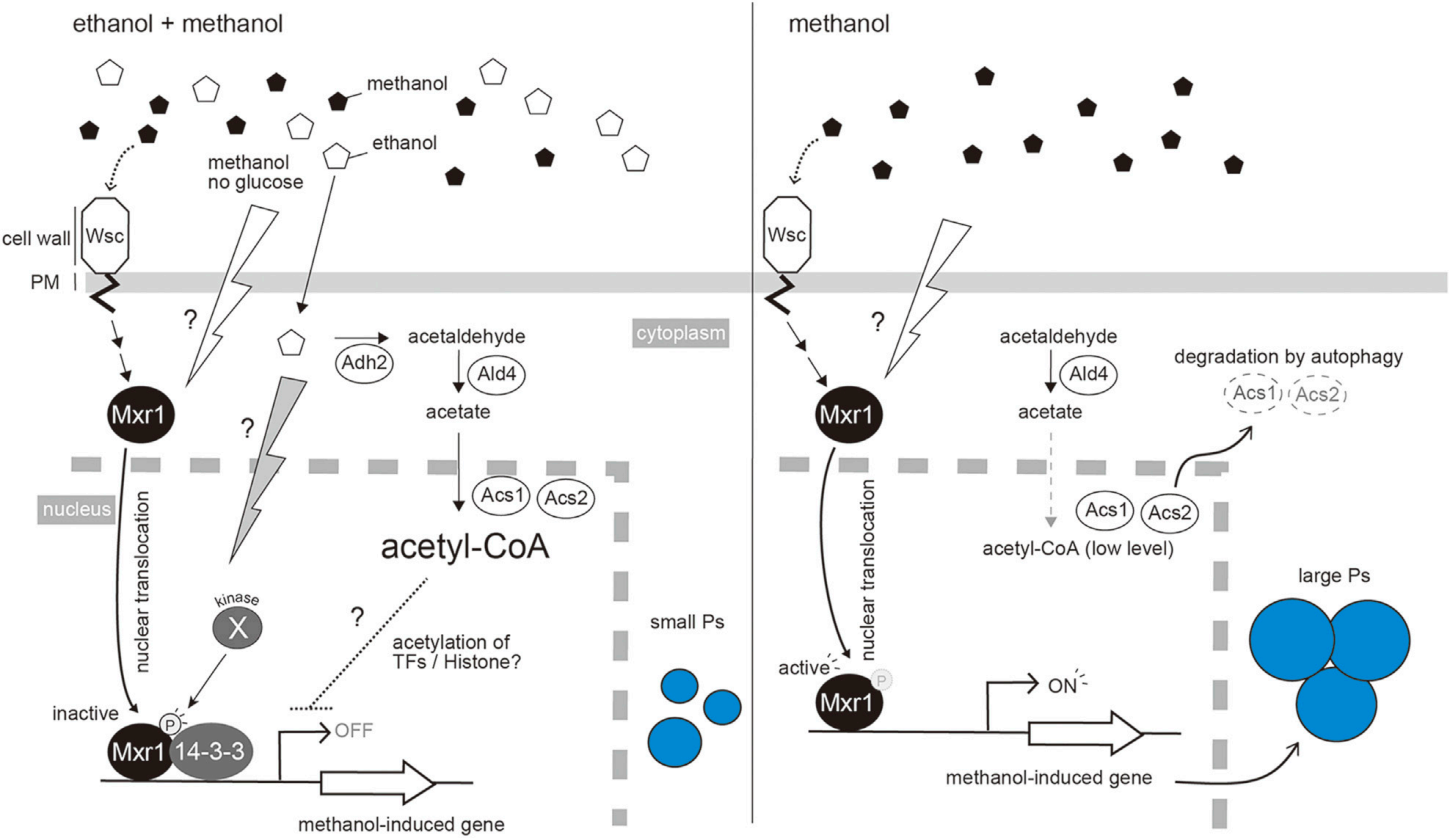
Repression of methanol-induced peroxisome proliferation by the co-presence of ethanol. Ethanol is metabolized to Acetyl-CoA by the sequential reactions catalyzed by Adh2, Ald4, Acs1 and Acs2. Acetyl-CoA then represses methanol-induced gene expression. As in *S. cerevisiae*, acetyl-CoA may be utilized for acetylation of histones or other transcription factors that repress the expression of methanol-induced genes (Takahashi et al., 2006). Mxr1 is a key transcription factor for methanol-induced gene expression. The presence of methanol, or removal of glucose, changes the localization of Mxr1 from the cytoplasm to the nucleus, where it can bind to the promoter regions of methanol-induced genes. In the presence of ethanol, an unidentified kinase inactivates Mxr1 by phosphorylation at the serine residue S215, which causes the interaction with 14-3-3 protein in the nucleus. This interaction inhibits the function of KpMxr1 activation domain (Parua et al., 2012) **(left panel)**. This process occurs independently of the acetyl-CoA pathway component. Changing to methanol media or depleting ethanol stimulates the dephosphorylated Mxr1 to activate the expression of methanol-induced genes. To release the ethanol repression effectively, Acs1 and Acs2 are degraded by autophagy **(right panel)**.

In *K. phaffii*, KpMxr1 is localized in the cytoplasm during glucose repression. However, during ethanol repression, KpMxr1 is translocated into the nucleus where it binds to the promoter regions of methanol-induced genes (*AOX1* and *DAS1*) but does not activate transcription. The phosphorylation of KpMxr1 S215 residue allows it to interact with 14-3-3 protein and inhibit the transcriptional activation by KpMxr1 (Parua et al., 2012) (Figure 2A). The study of binding site of KpMxr1 with 14-3-3 protein suggests that 14-3-3 protein acts to inhibit the function of KpMxr1 activation domain. Therefore, the activity of KpMxr1 is inhibited by different mechanisms in glucose and ethanol repression.

In addition to phospho-regulation of KpMxr1 during ethanol repression, our recent study demonstrated that synthesis of acetyl-CoA from ethanol by sequential reactions of alcohol dehydrogenase (ADH), aldehyde dehydrogenase (ALD) and acetyl-CoA synthetase (ACS) is involved in ethanol repression (Ohsawa et al., 2018). Amongst the deletion strains of *KpADH2* encoding alcohol dehydrogenase (*Kpadh2∆*), KpMxr1(S215A) mutant, and the double mutant [*Kpadh2∆*/KpMxr1(S215A)], the double mutant showed a higher-level release of ethanol repression than those with other single mutants. Thus, the effects of acetyl-CoA synthesis and phospho-regulation of S215 in KpMxr1 are additive, indicating that acetyl-CoA synthesis controls ethanol repression independently of KpMxr1. Further study is needed to reveal how acetyl-CoA and/or its metabolites control ethanol repression. In *S. cerevisiae,* a section of ACS localizes in the nucleus where it synthesizes acetyl-CoA, which acts as the substrates for acetylation of proteins such as histone (Takahashi et al., 2006). We assume that acetyl-CoA is utilized for acetylation of histones and/or transcription factors that are involved in ethanol repression (Figure 2).

ACS activity is decreased, on the transition from ethanol repression to methanol induction, and this is partially dependent on autophagic degradation (Ohsawa et al., 2018). Furthermore, overexpression of *KpACS1* suppressed the maximum level of methanol-induced gene expression. These results suggest that autophagic inactivation of ACS occurs to release ethanol repression effectively during methanol-induction (Figure 2B). Post-translational regulations, such as phospho-regulation and autophagic protein degradation, lead cells to regulate methanol-induced gene expression in a strict and effective manner.

## Repression of Pexophagy by Methanol Through the MAPK Pathway and by Atg30 Phosphorylation

Selective autophagy of organelles requires receptor proteins on the surface of the target organelle that recruit the autophagy-related (Atg) proteins to form autophagosomes (Xie and Klionsky, 2007). The receptor for pexophagy in *K. phaffii* is KpAtg30 (Farré et al., 2013 et al.). It interacts with the peroxins which are involved in peroxisome biogenesis (KpPex3 and KpPex14) and with three Atg proteins (KpAtg8, KpAtg11, and KpAtg37) (Farré et al., 2008; Farré et al., 2013; Nazarko et al., 2014; Burnett et al., 2015). Two phosphorylation events of KpAtg30 recruit Atg proteins. Phosphorylation sites of KpAtg30 are at serine residue S71 and S112 by an unknown kinase and KpHrr25, a homologue of casein kinase 1δ, respectively (Farré et al., 2013). The peroxisomal acyl-CoA-binding protein, KpAtg37, is recruited to the KpAtg30 in Pex3-dependnt manner. The recruitment of KpAtg37 to KpAtg30 enables binding of KpHrr25 to KpAtg30, leading the phosphorylation of KpAtg30 at S112 to recruit KpAtg11 to KpAtg30 (Zientara-Rytter et al., 2018). KpPex3 interacts with KpAtg30 at KpHrr25-binding site,_it makes a state that is unfavorable for KpHrr25 binding, leading a second phosphorylation at S71 to recruit KpAtg8 to KpAtg30. Our recent study provides further evidence of importance of phosphorylation in pexophagy; we demonstrated that KpWsc1 and its downstream MAPK negatively regulate pexophagy in the presence of methanol *via* repression of KpAtg30 phosphorylation (Ohsawa et al., 2021). Furthermore, KpRlm1-dependent genes encoding phosphatases (KpMsg5 and KpPtp2A) have a role in maintaining a low level of KpAtg30 phosphorylation in the presence of methanol (Ohsawa et al., 2021). In the *S. cerevisiae* CWI pathway, ScMpk1 activates ScRlm1 for induction of glucan synthase during cell surface stress (Rodicio and Heinisch, 2010; Levin, 2011). By analogy, we assume that KpWsc1 activates KpMpk1, and transmits a signal to KpRlm1 for suppression of pexophagy. As described above, the signaling pathway that controls peroxisome synthesis *via* the regulation of methanol-induced gene expression does not employ components of KpMpk1 and KpRlm1. These results suggest that the KpWsc1 methanol-sensing machinery regulates both the level of peroxisome biogenesis and the degree of pexophagy in response to the methanol concentration, by two distinct signaling pathways, MAPK-independent and MAPK-dependent pathways, respectively (Figure 1).

## Physiological Significance in the Phyllosphere

In the phyllosphere, the methanol concentration on plant leaves oscillates diurnally, being higher in the dark period and lower in the light period (∼0–0.3%), (Kawaguchi et al., 2011). The methylotrophic yeasts *C. boidinii* and *K. phaffii* can proliferate on plant leaves at a cell-division rate of 3-4 per 7–10 days (Kawaguchi et al., 2011). During proliferation on plant leaves, *C. boidinii* cells repeat the daily cycle of peroxisome biogenesis and degradation by pexophagy. During this diurnal cycle, factors for peroxisome biogenesis, methanol-utilizing enzymes, and pexophagy (e.g. CbAtg30) are essential for methylotrophic yeast cells growth. Evidently, they regulate peroxisome dynamics in response to the methanol concentration of the phyllosphere. We speculate that the methanol-sensing machinery, KpWsc1 and KpWsc3, are responsible for regulation of peroxisome homeostasis on plant leaves (Figure 1). Remarkably, non-selective (bulk) autophagy, which is dependent on Atg1 but not Atg30, occurs throughout the daily dark-light cycle (Kawaguchi et al., 2011). Conversely, Atg30-dependent pexophagy is only observed in the light period. The negative regulation of pexophagy *via* methanol-sensing machinery (Wsc1 and MAPK) could explain how pexophagy is selectively inhibited during the dark period (when the methanol concentration is higher), while the non-selective autophagy machinery is active throughout the daily light-dark cycle. Revealing the molecular mechanism of post-translational regulation of methanol-induced gene expression and peroxisome dynamics would facilitate the improvement of the system for heterologous gene expression and would also further our understanding of the physiological roles of these regulatory machineries as survival strategies in the phyllosphere.

## Concluding Remarks

Peroxisomes are highly dynamic organelles whose homeostasis is controlled by biogenesis and pexophagy in response to the extracellular environment. In the methylotrophic yeast *K. phaffii*, huge peroxisomes are induced during growth on methanol and subsequently degraded by pexophagy in response to both the concentration of methanol or to a change in the carbon source (glucose and ethanol). Although the molecular mechanism of peroxisome dynamics has been studied in detail, less is known about the upstream signaling pathway and those factors responsible for regulating these peroxisome dynamics at a physiological level. The methanol-signaling pathway mediated by methanol-sensing machinery of WSC family proteins has been shown to regulate peroxisome dynamics depending on the environmental methanol concentration through methanol-induced gene expression and the phospho-regulation of a pexophagy receptor, Atg30. Based on these results, we propose that the physiological significance of peroxisome homeostasis is linked to the diurnal oscillation in methanol concentration on plant leaves. We also show new molecular machinery responsible for ethanol repression of the peroxisome induction, by acetyl-CoA. Further studies on the signaling mechanisms involved in peroxisome homeostasis will facilitate a better understanding of the physiological role of the regulation of peroxisome dynamics.
